# Notices

**Published:** 1864-02

**Authors:** 


### NOTICES.

"Golden Stories," published by Mr. Wood, 61 Walker street, embrace five delightful little books, in uniform binding, for young children: "The White Kitten," "The Tent in the Garden," "Loving Words or Loving Deeds," "The Water-Melon," and "Willie Wilson, the Newsboy." This House, it will be remembered, has had for many years the most extensive assortment of medical publications of any other establishment in the country, besides a large collection of miscellaneous books, all of sterling value.

Almanac for 1864. Being the Tenth Illustrated Register of Rural Affairs. By Luther Tucker & Co., Albany, N. Y. Sent free by mail for twenty-five cents, giving a vast amount of useful, reliable, and practical information to farmers upon every variety of subject connected with the cultivation of land.

The Mother's Journal, monthly, $1 a year, New-York, by Mrs. Caroline O. Hiscox, is filled with articles of sterling value; the selections are made with a wise discrimination, nothing frivolous ever appears in its fair pages. It merits a very general circulation.

"It Beats the World," said our good-natured old colored laundress the other day, in admiration of the "Universal Clothes-Wringer," and her reasons were terse and laconic: "It saves work and clothes too."

Weather Indicator. Charles Wilder, of Peterboro, N. H., manufactures Woodruff's Barometer, which combines in a remarkable degree cheapness, accuracy, simplicity, durability, and portability. Prices from $5 to $20.

Photographic Magnifier, a most charming accompaniment to photographic albums. $1, $1.50 and $3.

The Photographic Magnifier affords the sweetest of all pleasures, as often as used for inspecting the portraits of those dear to us. Sold by P. C. Godfrey, 831 Broadway, New-York.

To Youth.—A warning against advertisements headed Physical Debility. Confessions of an Invalid. Marriage Guide. Warning to Young Men. Manhood Restored. Essence of Life. Advice to the Married. Early Indiscretions. Loss of Memory. Nervous Debility. See Hall's Journal of Health for December, 1863. Sent post-paid for twelve cents.

Personal.—Our office for medical consultation temporarily, is at 831 Broadway, New-York, from eleven to one o'clock, daily. Ladies who desire a consultation are requested to notify us, when we will appoint a specific hour. Gentlemen who can not be in the city during the above hours can arrange a special appointment. We do not desire interruption at present, except from eleven to one, unless necessary.

The last editions of "Health and Disease" and "Sleep" are exhausted; new editions will be printed about first of February.

Any one of our books will be sent post-paid to any person sending us four new subscribers.

John G. Broughton, Esq., No. 13 Bible House, New-York, has sent us four publications of the American Tract Society, No. 28 Cornhill, Boston. "Temperance Tales," by Lucius M. Sargent, is an invaluable book for all classes, but especially for the young. "Pleasant Tales," in prose and verse, with twenty-six engravings. Contents: Mark's Temptation; Bill and his Bible; A Lesson from the Birds; True Courage, and fifty-four ! other useful and most interesting stories, sent for forty cents; also, "Black and White," by Mrs. Jane D. Chaplin, which will find thousands of admiring and sympathizing readers. Christ the Children's Guide, by Rev. J. S. Sewall, a sweetly instructive little book of thirty-six pages.

